# Sexual and reproductive health impacts of modern slavery and human trafficking: a scoping review protocol

**DOI:** 10.1136/bmjopen-2026-122246

**Published:** 2026-10-07

**Authors:** Lauren Eglen, Katherine Watson, Nicola Wright

**Affiliations:** 1 University of Nottingham, Nottingham, UK; 2 Research Fellow in Reproductive Justice and Anti-Trafficking, University of Nottingham, Nottingham, UK; 3 School Health Sciences, University of Nottingham, Nottingham, UK

**Keywords:** Health, Systematic Review, Gender-Based Violence

## Abstract

**Abstract:**

**Introduction:**

Modern slavery and human trafficking (MS-HT) are urgent and increasing global issues that violate human rights and have long-lasting consequences for those affected. The impact on individuals’ health is well-documented, and MS-HT are recognised as critical public health issues, reflecting the extensive physical, psychological and social harms they produce, as well as the essential role of health systems in prevention, identification and response. Despite this, the sexual and reproductive health (SRH) impacts of exploitation in these contexts remain insufficiently addressed and understood. This scoping review will explore the SRH needs, outcomes and challenges of those impacted by MS-HT by synthesising existing evidence. This review is the first phase of a large-scale study to develop a reproductive justice framework for MS-HT research and practice towards improving access to SRH services.

**Methods and analysis:**

This scoping review will follow Arksey and O’Malley’s framework which consists of the following five steps: (1) identifying the research question(s); (2) identifying relevant studies; (3) study selection; (4) charting the data; (5) collating, summarising and reporting the results. We will also follow the Joanna Briggs Institute and the Preferred Reporting Items for Systematic Reviews and Meta-Analyses extension for Scoping (PRISMA-ScR) guidelines for a scoping review. We will include English language peer-reviewed publications and grey literature on the SRH needs, outcomes and challenges of those impacted by MS-HT published from January 2000 to August 2026. The three-step search strategy will involve an initial search of eight databases, citation mining of previous review studies, an advanced Google search and systematic searches of key anti-trafficking and SRHR organisation repositories. We will search EMBASE (Ovid), MEDLINE (Ovid), PsycINFO (Ovid), Scopus, Maternity and Infant Care (MIDIRS), CINAHL Ultimate, HeinOnline and Web of Science and the websites for the WHO, Human Rights Watch, Anti-Slavery International, International Justice Mission, Amnesty International, Guttmacher Institute and UN Women. Two reviewers will screen the titles, abstracts and full texts of publications against the inclusion/exclusion criteria. Data will be extracted using a customised data charting form in Excel developed by the research team. Extracted data will be analysed using narrative analysis. Searches will be run starting 1 September 2026.

**Ethics and dissemination:**

Ethical approval is not required for a scoping review given that it is a synthesis of publicly available publications. Dissemination will be through publication in a peer-reviewed journal and presentation at relevant conferences, convenings of policymakers and civil society organisations, including those currently collaborating with the University of Nottingham’s Rights Lab.

**STRENGTHS AND LIMITATIONS OF THIS STUDY**

- This multidisciplinary research team has expertise across human trafficking and modern slavery, sexual and reproductive health and rights and health services.
- Three guidelines for conducting scoping reviews were followed to maximise rigour and reproducibility.
- The review includes both peer-reviewed and grey literature to capture academic and broader findings.
- A key limitation is that only papers in English will be included due to resource limitations for translation, meaning potential exclusion of relevant publications in other languages.

## Introduction

 Modern slavery and human trafficking (MS-HT) are significant global challenges that violate fundamental human rights. While difficult to know the true prevalence of these crimes due to their clandestine nature, recent global estimates suggest that the number of people impacted globally is on the rise. Human trafficking is the recruitment, transportation, transfer, harbouring or receipt (‘the act’) of people through force, fraud or deception (‘the means’) for the purpose of exploitation. As defined by the UN Protocol to Prevent, Suppress and Punish Trafficking in Persons Especially Women and Children, exploitation shall include, at a minimum, the exploitation of the prostitution of others or other forms of sexual exploitation, forced labour or services, slavery or practices similar to slavery, servitude or the removal of organs.^1^ In 2024, the United Nations Office for Drugs and Crime reported a 25% increase in detected victims of human trafficking in 2022 compared with 2019, with a total of 202 478 detected victims between 2020 and 2023.^2^ Of these, 62% were adults and 39% were children, with women and girls comprising 61% of detected victims. Detected forms of exploitation included forced labour (42%), sexual exploitation (36%), forced criminality (8%), mixed forms of exploitation (8%), forced begging (1%), forced marriage (1%) and other purposes (4%).

The parameters of modern slavery continue to be debated among scholars, policymakers and practitioners. This review employs the term as an umbrella for the different severe forms of exploitation, which are otherwise legally defined.^3^ In this definition, modern slavery includes forced labour, including domestic servitude, bonded labour, sexual exploitation, slavery and institutions and practices similar to slavery and forced marriage. The most recent global estimates on modern slavery suggest that, on any given day in 2021, approximately 49.6 million people were living in conditions of modern slavery—an increase of 10 million from previous estimates—including 27.6 million in forced labour and 22 million in forced marriage.^4^

MS-HT are recognised as critical public health issues, reflecting the extensive physical, psychological and social harms they produce, as well as the essential role of health systems in prevention, identification and response. Individuals exploited in MS-HT are reported to experience a range of physical and mental health outcomes as a result of adverse living and working conditions and exposure to physical, sexual and emotional violence.^5^ Physical health problems reported include physical injury, headaches, fatigue, dizziness, back pain, memory problems, stomach pain, pelvic pain, gynaecological infections, weight loss, lesions or warts, unwanted pregnancies and abortions.^6^ Those impacted by human trafficking also experience significant mental health problems including depression, anxiety, PTSD and complex PTSD, suicidal thoughts and attempted suicide, as well as substance abuse.^6–8^ Individuals trafficked for sexual exploitation in particular have been identified as being at higher risk of adverse sexual health outcomes, including sexually transmitted infections (STIs) and HIV/AIDS, as well as becoming pregnant during exploitation and experiencing either voluntary or forced abortions.^9^ While healthcare settings are considered to be key points for intervention in modern slavery and human trafficking, the totality of control exerted over individuals while in situations of exploitation restricts access to healthcare and healthcare services, meaning that conditions often go untreated, and exploitation goes un-identified.

Sexual and reproductive health (SRH) remains a central global health priority. Sexual and reproductive health is understood holistically and from a rights-based perspective; this review adopts the Guttmacher-Lancet Commission’s 2018 definition which integrates international agreements, WHO publications and human rights treaties and principles: ‘Sexual and reproductive health is a state of physical, emotional, mental and social wellbeing in relation to all aspects of sexuality and reproduction, not merely the absence of disease, dysfunction or infirmity.’^10^ The Guttmacher-Lancet Commission on Sexual and Reproductive Health and Rights further characterises SRH as an ‘unfinished agenda’, highlighting persistent and widespread gaps in access to essential services.^10^ Each year in developing regions, more than 30 million people give birth outside of health facilities, over 45 million receive inadequate or no antenatal care and more than 200 million people who wish to avoid pregnancy are not using modern contraception.^10^ The Commission further estimates that up to 25 million unsafe abortions occur annually, with significant implications for morbidity and mortality. In addition, approximately 350 million individuals require treatment for one of the four curable sexually transmitted infections, and nearly 2 million people acquire HIV each year. SRH scholarship has emphasised the importance of intersectionality and the social determinants of health, calling for health systems that are responsive to complex and overlapping vulnerabilities, including trafficking.^11 12^

Individually, MS-HT and the achievement of universal SRH represent urgent global challenges, marked by profound inequities and unmet needs. Despite clear points of convergence, their intersection remains fragmented and insufficiently understood. Extant reviews on the health impacts of MS-HT have largely focused on health screening and system responses, as well as reviews focused specifically on the mental health outcomes for survivors.^13–20^ Reviews relevant to the SRH outcomes of those impacted by modern slavery and human trafficking have largely focused on the sexual health impacts of commercial sexual exploitation, most specifically risk of STIs and HIV/AIDS, and on maternity care.^21–24^

This scoping review represents the first phase of a large-scale study to develop a reproductive justice framework for MS-HT research and practice. Findings will be used in the next stage to inform data collection, including drafting key informant interview frameworks to be carried out with activists, community organisations and healthcare professionals, and the co-development of participatory photography workshops with impacted populations. The resulting data produced from interviews and participatory workshops will be analysed to produce a better understanding of the SRH needs, risks and outcomes of those impacted by MS-HT, including analysis of how interlocking systems of oppression and intersecting social identity shapes access to healthcare services, towards improving education and awareness among healthcare professionals and enhancing the sexual and reproductive healthcare provided to survivors.

## Methodology and analysis

A scoping review was selected for its suitability in addressing complex topics that draw on diverse study designs, as well as its capacity to synthesise and map concepts that have not yet been comprehensively examined.^25^ Given that no prior review has explored comprehensively the intersections of MS-HT and SRH, this approach is particularly appropriate. This review will employ the robust scoping review framework proposed by Arksey and O’Malley following five stages:

1. Identifying the research question(s).
2. Identifying relevant studies.
3. Study selection.
4. Charting the data.
5. Collating, summarising and reporting the results.

The review will be reported in alignment with the Preferred Reporting Items for Systematic Reviews and Meta-Analyses for Scoping Reviews guidelines (PRISMA-ScR) and with Joanna Briggs Institute guidance for scoping reviews.^26^

### Identifying the research questions

Arksey and O’Malley highlight the iterative nature of scoping reviews, suggesting that decisions about setting parameters for a large volume of bibliographic references can be made once there is a sense of the scope of the field of research. At the same time, it is crucial to start out with research questions to guide the review. This review will be guided by the following questions.

#### Primary question

What are the impacts on sexual and reproductive health of modern slavery and human trafficking?

#### Secondary research questions

- What are the primary SRH issues faced by people who experience MS-HT?
- Which services do they require to address their SRH issues?
- What unique challenges do people who experience MS-HT face in accessing SRH services and information?
- How do these differ across different populations and contexts of MS-HT?

##### Identifying relevant studies

The search strategy was developed by the research team with expertise in sexual and reproductive health and rights and human trafficking, in consultation with a librarian at the University of Nottingham. This will comprise a search of eight electronic databases; advanced Google searches; systematic search of key non-governmental organisations/inter-governmental organisations (NGO/IGO) repositories; citation mining; and targeted outreach to academics, practitioners and civil society organisations. The following electronic databases will be searched from 2000 to present day.

- EMBASE (Ovid).
- MEDLINE (Ovid).
- PsycINFO (Ovid).
- Scopus.
- Maternity and infant care (MIDIRS).
- CINAHL Ultimate.
- Hein Online.
- Web of Science.

The search strategy uses key search terms related to SRH (eg, pregnancy, abortion, sexually transmitted infections) combined with key search terms for MS-HT, using Boolean operators (‘AND’ and ‘OR’). Search terms were tested and refined over multiple iterations with consideration of the purpose and scope of this review, as well as the nature of scoping reviews. To limit the risk of increasing specificity at the expense of sensitivity and narrowing the search logic, search terms for specific contexts with a higher risk of MS-HT were not included. And while there are specific sectors within which MS-HT are at higher risk of occurring, sector-specific terminology is inconsistently applied in MS-HT literature which variously identifies exploitation according to industry, occupation, activity, commodity and/or supply chain. To ensure a broad range of results with regard to forms of exploitation, search concepts focused on legally defined and/or widely recognised forms of MS-HT as laid out in the United Nations Palermo Protocol and the International Labour Organization and Walk Free Foundation’s Global Estimates of Modern Slavery. This included key search terms for trafficking and modern slavery for exploitation in forced labour, including sexual exploitation, bonded labour (including debt bondage) and domestic servitude, as well as for forced marriage and for slavery.

Search terms will be appropriately modified for each database, and the reference lists of relevant scoping and systematic reviews will be screened for additional relevant studies. Researchers also consulted with project partners made up of survivor-led anti-trafficking and gender-based violence organisations in focus countries when developing the search strategy. Potential focus areas were discussed, and key issues and terms were identified based on the SRH issues being seen in MS-HT contexts in their work.

Advanced Google searches will be conducted using the same terms, using incognito mode to minimise past search bias. The first 10 pages of results will be screened. This will allow for the screening of relevant grey literature, including non- and inter-governmental organisation reports and policy reports. In addition, a systematic search of key NGO/IGO repositories will be conducted to screen for additional relevant grey literature. We will search the websites of WHO, Human Rights Watch, Anti-Slavery International, International Justice Mission, Amnesty International, Guttmacher Institute, UN Women, the International Labour Organization, Focus on Labour Exploitation, Walk Free Foundation and the International Organization for Migration.

Finally, online searches will be complemented by targeted outreach to key academics, practitioners and community service organisations. Authors of relevant systematic and scoping reviews will be contacted with an explanation of the scoping review and a request for relevant literature. In addition, academics and practitioners in the three study countries engaged in the Wellcome Trust-funded research project (UK, Kenya and South Africa) will be contacted with the same request.

The complete Ovid Medline search strategy is available in the online supplemental appendix. All search strategies, exactly as run, will be made available on publication of the final review.

The scoping review protocol has been registered on Figshare and is available at: https://doi.org/10.6084/m9.figshare.33209307.

### Study selection

The eligibility criteria for this review are found in table 1 based on the population, concept and context framework (Pollock *et al*).^27^

**Table 1:** Inclusion and exclusion criteria

Criteria	Included	Excluded
Year published	From January 2000 to August 2026	Before January 2000 or after August 2026
Language	English	All others
Type of paper	Empirical and non-empirical studies with extractable data	Other types of papers without extractable data (eg, commentaries)
Population	Papers that focus on people who have experienced trafficking and/or modern slavery, or those that disaggregate results for people who have experienced trafficking and/or modern slavery	Papers that do not focus on people who have not experienced trafficking and/or modern slavery; papers that include those who have experiences of trafficking and/or modern slavery but do not disaggregate their experiences from those of other populations (eg, migrants or sex workers)
Concept: SRH	Papers that address SRH outcomes and issues as well as the barriers faced in accessing SRH services, for example abortion, contraception and treatment for sexually transmitted infections	Papers that do not address SRH outcomes, issues or barriers
Context	Papers that address SRH in contexts of human trafficking and recognised forms of modern slavery	Papers that do not address SRH in contexts of human trafficking and recognised forms of modern slavery

SRH, Sexual and reproductive health.

Studies published from January 2000 to August 2026 will be included for review, reflecting the adoption of the United Nations’ Palermo Protocol which established an internationally recognised definition of human trafficking. Where papers present data across multiple populations, one or more of which is people who have experienced modern slavery and human trafficking, the papers will be included only where data relevant to the people with experience of MS-HT can be disaggregated. This includes, for example, studies that include survivors of human trafficking within broader gender-based violence and violence against women and girls populations, but where the specific experiences of MS/HT survivors are not detailed.

Further, the focus of this scoping review is on understanding the SRH issues faced by people who have experienced MS-HT and the services that they need. As such, studies that focus on the experiences of medical professionals in providing services will only be included where they include extractable data related to the SRH outcomes and issues faced by those who have been exploited, as well as the barriers they face in accessing SRH services. As the focus of this scoping review is on the SRH needs of those with lived experience rather than the preparedness of service providers to meet that need, studies focused on assessing levels of healthcare provider knowledge and awareness, and on developing healthcare provider training and guidance for identification of MS-HT will not be included.

Given the review’s focus on the SRH needs and outcomes of those who have experienced MS-HT across intersecting identity factors, there will be no exclusion criteria based on factors such as age, race, gender identity, class, sexuality, ethnicity, religion, etc of those with lived experience. The object of study is broadly those with lived experience of MS-HT and, as such, there are also no specific inclusion criteria across these intersecting identity factors.

Finally, no geographical limits will be applied, though these criteria may be revised post-hoc based on our increasing familiarity with the literature. Only studies published in English will be included. This is a key limitation of this study due to a lack of resources available for accurate and reliable translation of studies not published in English.

### Charting the data

Reference management software will be used to organise all identified articles, and duplicates will be identified and removed. Articles will be independently screened against the inclusion/exclusion criteria by two reviewers at the title/abstract level for inclusion in full-text review. Dual independent screening of full-text returns for review will then be conducted by two reviewers. At both title/abstract and full-text review stages, any disagreements on inclusion/exclusion will be resolved through discussion to reach consensus. The selection process will follow the recommendations in the Preferred Reporting Items for Systematic Reviews and Meta-Analyses Extension for Scoping Review (PRISMA-ScR) checklist and the Preferred Reporting Items for Systematic Reviews and Meta-Analyses (PRISMA) flow chart diagram for reporting items for systematic reviews will be used to demonstrate the process of articles screening, inclusion and exclusion.

A customised data charting form in Excel will be developed, and data extraction from up to 10 papers will be tested by two authors and modified where needed before charting moves forward. The remaining papers will be fully charted by one author and verified by a second author. The charted data will include standardised categories applied to all reviewed papers (see table 2), though this list will be refined iteratively as familiarity with the literature increases.

**Table 2:** Data charting categories, sub-categories and descriptions

Main category	Sub-categories	Description
Bibliographic information	Title Author(s) Publication type Journal Research field Year	The ‘research field’ sub-category will allow tracking of the various fields of study that address the topics of SRH and MS-HT.
Geography	Region of study Country or countries of study	This will be tracked to understand the regions with the heaviest concentration of research and evidence on the scoping review topics.
Study population		The sub-categories for this category will be developed iteratively following review of the included studies.
Study context	Form of MS-HT Sector/industry	The sub-categories for this category will be developed iteratively and relate to the type of MS-HT to which the evidence relates.
Aim of study		
Study design	Qualitative Quantitative Mixed methods Scoping review Systematic review	Other sub-categories will be added iteratively with review of the included studies.
Study limitations		These will be as stated by the authors in each of the papers, to contextualise findings and their generalisability.
SRH issue	Pregnancy Contraception Abortion Sexually transmitted infections HIV	Other sub-categories will be added iteratively with review of the included studies.
Key findings		Up to three key findings will be extracted and charted from each of the included papers.
Study implications		This includes any policy or programmatic implications stated in the paper.
Reviewer’s comment		This category is a space for the reviewer to place notes for further consideration during the analysis and writing or for further research.

MS-HT, modern slavery and human trafficking; SRH, sexual and reproductive health.

No critical appraisal will be completed as per the aim of scoping reviews to identify and summarise the available evidence rather than provide an assessment, consistent with the methodology used.

### Collating, summarising and reporting the results

After extracting data, a narrative synthesis will be conducted to synthesise findings of the different studies. Due to the potential range of studies that may be included in this scoping review, a narrative synthesis is the most appropriate way to synthesise the findings.

Popay *et al* outline steps for conducting a narrative synthesis for systematic reviews: (1) developing a theory of how the intervention works, why and for whom; (2) developing a preliminary synthesis, (3) exploring relationships within and between studies, and (4) assessing the robustness of the synthesis overall.^28^ Step 1 is most appropriate for reviews that establish a theory of change at an earlier stage; this step will not be included. However, Steps 2 to 4 will guide the approach to synthesising data for this scoping review. The included literature will be organised for preliminary synthesis in a manner that allows for patterns to emerge. This may include inductive thematic coding. Next, relationships between studies will be interrogated using the preliminary analysis to explain differences and similarities. This will include interrogation of varying study designs, contexts and populations. Once this is completed, an assessment will be made of the robustness of the existing research to inform the reporting of results.

Results will be reported in two different ways. First, numerical analysis will be conducted and will include the extent, nature and distribution of the studies on SRH and MS-HT. This will be used to ‘shed light on the dominant areas of research in terms of intervention type, research methods and geographical location’, which will give an overview of the dominant areas of research that exist and the corresponding gaps. Second, the data will be analysed thematically. The thematic framework may arise organically during Stage 4 of the scoping review process and may, for example, be organised around study population, study context and/or SRH issue. It will be refined during the abovementioned preliminary synthesis of the literature.

At the time of submission of this protocol, the preparation stage has been completed, including the development of research objectives and identification of questions for the scoping review, formulation of the search strategy and finalisation of the inclusion/exclusion strategy. The study is now ready to begin the review process, starting with the search of relevant literature across the selected databases on 1 September 2026. The planned end date for the scoping review is 31 December 2026. This timeline considers the anticipated availability of relevant literature and allows for completion of all review stages: systematic searching, screening, data extraction, narrative synthesis and manuscript preparation.

## Ethics and dissemination

Ethical approval is not required for a scoping review given that it is a synthesis of publicly available publications. Dissemination will be through publication in a peer-reviewed journal and presentation at relevant conferences, convenings of policymakers and civil society organisations, including those currently collaborating with the University of Nottingham’s Rights Lab.

## Supplementary material

10.1136/bmjopen-2026-122246 online supplemental file 1
