# Supplementary material for: Sexual and reproductive health impacts of modern slavery and human trafficking: a scoping review protocol
Source: BMJ Open. 2026 Oct 7;16(10):e122246. doi: 10.1136/bmjopen-2026-122246 (PMC13647010; doi:10.1136/bmjopen-2026-122246)
Supplement: Supplementary file 1 [file bmjopen-2026-122246.reviewer_comments.pdf]

PEER REVIEW HISTORY

BMJ Open publishes all reviews undertaken for accepted manuscripts. Reviewers are asked to complete a checklist review form and are provided with free text boxes to elaborate on their assessment. These free text comments are reproduced below.

ARTICLE DETAILS

Title (Provisional)

The sexual and reproductive health impacts of modern slavery and human trafficking: A scoping review protocol

Authors

Eglen, Lauren; Watson, Katherine; Wright, Nicola

VERSION 1 - REVIEW

**Reviewer**	**1**
**Name**	**Stoklosa, Hanni**
**Affiliation**	**Brigham and Women's Hospital, Massachusetts General Hospital, Emergency Medicine**
**Date**	**07-Jul-2026**
**COI**	**None**

Overall, this is a clearly written and timely protocol for a scoping review mapping the intersection of sexual and reproductive health (SRH) with modern slavery and human trafficking (MS-HT). A scoping review is an appropriate and well-justified design, the reproductive-justice and intersectionality framing is a genuine strength.

The following are areas I would flag for consideration.

1. Single-reviewer screening and extraction limits reliability. The protocol states that title/abstract screening will be done by one reviewer, with a second reviewer checking only "20% or 250 returns, whichever is lower," and that full-text dual review and data-charting calibration apply only to the "first 10% or 20" papers. Because of the "whichever is lower" caps, the proportion independently double-screened could become very small as the volume of records grows, which is a recognised source of error in evidence synthesis. Current JBI and PRISMA-ScR best practice favours independent dual screening of all titles/abstracts and full texts. I would ask the authors either to commit to fuller independent double-screening or, if resource constraints preclude this, to justify the chosen approach explicitly and describe how screening disagreements and single-reviewer error will be mitigated (e.g., conflict resolution procedure, a larger calibration sample, or use of a third reviewer).

2. Protocol registration is not reported. PRISMA-ScR item 5 (Protocol and registration) appears unaddressed — the authors' own checklist leaves this entry blank. Please register the protocol and state the registration location/number, or explain why registration was not undertaken.

3. Planned study timeline is not stated. The manuscript gives only the literature-coverage window (2000–2026) and no projected timeline for the review itself. I recommend adding the planned study dates (anticipated start and completion of searching, screening and synthesis) and to confirm that data collection is not yet complete.

4. Exclusion of provider-focused studies may conflict with the stated aims. Studies focusing on the experiences of medical professionals in providing services are excluded. However, secondary question 2 asks "Which services do they require…?" and the wider programme aims to improve education and awareness among healthcare professionals. Provider-facing literature is often where barriers to SRH service delivery and unmet need are documented. Please clarify the rationale for this exclusion and consider whether evidence on service provision/barriers reported in provider-focused studies (where it speaks to survivors' needs) might be retained, or acknowledge the resulting evidence gap as a limitation.

5. Framing of SRH as a "domain of human suffering" is conceptually inconsistent. The opening sentence states that "modern slavery and human trafficking (MS-HT) and sexual and reproductive health (SRH) represent vast and urgent domains of human suffering." This parallelism treats the two as the same kind of object, but they are not symmetrical: MS-HT is itself a harm and rights violation, whereas SRH is a domain of health and rights in which the suffering arises from inequities and unmet need rather than from the domain as such. More importantly, this framing is internally inconsistent with the manuscript's own working definition: one paragraph later the authors adopt the Guttmacher–Lancet definition of SRH as "a state of physical, emotional, mental, and social wellbeing… not merely the absence of disease, dysfunction, or infirmity." Describing a domain of wellbeing as a domain of suffering both contradicts that definition and weakens the rights-based framing the authors are establishing. I suggest retaining "suffering" for MS-HT and recasting SRH in the language of need/inequity. For example, "Modern slavery and human trafficking represent a vast domain of human suffering and rights violation, while sexual and reproductive health represents an equally urgent global priority marked by profound inequities and widespread unmet need."

6. Cross-disciplinary and grey-literature coverage may be too narrow for a nascent field. Five of the six databases are predominantly biomedical or nursing in scope (EMBASE, MEDLINE, PsycINFO, MIDIRS, CINAHL), with Scopus the only broadly multidisciplinary source, and Scopus's coverage of legal scholarship in particular is limited. Because the health & trafficking field is at an early stage, a substantial and often formative share of the relevant evidence sits outside the health literature, in legal and human-rights scholarship, criminology, the social sciences, and grey literature (NGO/IGO and policy reports). Health-database-restricted searches systematically under-capture this material, and the appendix

strategy — built largely on Emtree/MeSH headings — will not translate onto non-health indexes. The protocol partially mitigates this through advanced Google searches, citation mining and targeted expert outreach, which is commendable. I would nonetheless recommend adding at least one legal index (e.g., HeinOnline or Index to Legal Periodicals) and one social-science/criminology index (e.g., Web of Science Social Sciences Citation Index, Sociological Abstracts/ASSIA, or Criminal Justice Abstracts), with search terms adapted for each, and expanding the grey-literature protocol beyond the first ten pages of Google to include systematic searching of key NGO/IGO repositories.

7. Search sensitivity to labour trafficking and forced labour. The existing SRH-and-trafficking literature is heavily weighted toward sexual exploitation, while labour trafficking, forced labour, domestic servitude and bonded labour remain comparatively neglected despite documented SRH consequences (e.g., pregnancy and reproductive coercion among domestic and agricultural workers, menstrual and occupational reproductive health, and barriers to care). Given this pre-existing imbalance in the evidence base, I would encourage the authors to deliberately err toward over-inclusion of labour-exploitation contexts, in both the search and screening. Two concrete points. (i) In the Ovid MEDLINE strategy, the trafficking proximity clause — "(human* or sex* or people or person* or wom#n* or female* or child* or marriage) adj3 traffic*" — omits labour, so records emphasising "labour/labor trafficking" or "labour exploitation" without co-located "human trafficking" phrasing may be missed. Authors could consider adding labo?r* to that clause, together with terms such as "forced labo?r", "labo?r exploitation", "indentured", "peonage", "involuntary servitude", and potentially high-risk sector terms (domestic, agricultural, fishing, construction work). (ii) Because SRH terms map more readily onto sexual-exploitation contexts, requiring both concepts to be central risks dropping labour-trafficking papers where SRH is present but incidental; a generous SRH block (including, e.g., menstrua*, "reproductive coercion", and maternal/obstetric terms) is advisable.

8. Gender-inclusive terminology for pregnancy and reproduction. In their own narrative the authors use gendered language for reproduction including "women who wish to avoid pregnancy," "women and girls," "becoming pregnant during exploitation" and do not use inclusive formulations such as "pregnant people" or "people who can become pregnant." This is somewhat inconsistent with the reproductive-justice framing, the explicit decision to set no exclusion criteria on gender identity or sexuality, and indeed one of the authors' own cited sources (ref 27, "Maternal Health Concerns for Pregnant Labor-Trafficked People").

9. Tension between "no geographical limits" and English-only inclusion. English-only inclusion is a reasonable, but it sits in some tension with the "no geographical limits applied". English-only inclusion will systematically under-represent high-prevalence non-Anglophone contexts.

10. Patient and public involvement. PPI is reported as none. For a study explicitly grounded in reproductive justice and lived experience, and one whose later phases involve

participatory photography with affected populations, the absence of PPI at the protocol stage is worth a justification, or consideration of involvement (e.g., survivor or community-organisation input on search terms/charting categories).

VERSION 1 - AUTHOR RESPONSE

Reviewer 1 comments:

1. Single-reviewer screening and extraction limits reliability: We have re-evaluated the capacity of researchers on the team and have committed to independent double screening of title, abstract and full-text returns. This is detailed in the revised protocol.
2. Protocol registration is not reported: We have resistered the protocol on Figshare and have provided the registration number in the revised protocol.
3. Planned study timeline is not stated: We have added the updated planned study timeline to the revised protocol. We initially planned to run searches upon acceptance of the protocol and as such did not have specific dates in mind. This has now been amended and we will run searches on 1st September 2026.
4. Exclusion of provider-focused studies may conflict with the stated aims: We understand the reviewers point here regarding the experiences of medical professionals including data on the services those with lived experience of MS-HT require. This was originally excluded as we were focused in specifically on the perspective of those with lived experience and only studies which included lived experience perspectives. We have taken the reviewer's feedback on board and have revised our inclusion criteria to include studies on the experiences of medical professionals providing services but only if they include extractable data related to the RH outcomes and issues of those who have been exploited, as well as their barriers in accessing services. Publications focused on levels of knowledge, awareness, and potential training programmes for healthcare professionals will not be included. While we recognise that a lack of knowledge and awareness among healthcare professionals exists as a barrier to services, and this will be included where it arises in included returns, we believe that studies focused on assessing HCP knowledge and awareness and those putting forward potential trainings address a related but separate issue to our focus on the sexual and reproductive health issues faced by those with experience of exploitation in modern slavery and human trafficking. We have updated the revised protocol to reflect the expansion of the inclusion criteria.
5. Framing of SRH as a "domain of human suffering" is conceptually inconsistent: Thank you to the reviewer for catching the conceptual inconsistency in the framing of this sentence. The framing has been edited to ensure it is clear we are positioning both combatting MS-HT and achieving universal SRH are urgent global challenges. Separately, we have framed t MS-HT as a violation of human rights and SRH as a global health priority. We hope that our edits have addressed this piece of feedback adequately.
6. Cross-disciplinary and grey-literature coverage may be too narrow for a nascent field: We take the reviewers concerns regarding the medical-heavy focus of chosen databases and have expended our search to include HeinOnline and Web of Science. Web of Science was not originally included due to consultation with a university librarian advising that Scopus and Web of Science covered largely the same scholarship and as such there was no need to include both in searches. However, after looking further into the subject areas of both databases we can see the benefit of including both social science databases and have amended

our search strategy accordingly. We have also expanded our grey-literature search to include the systematic searching of key anti-trafficking, anti-slavery and SRHR NGO/IGO repositories and have detailed these in the revised protocol.
7. Search sensitivity to labour trafficking and forced labour: We take the reviewer's point regarding the weighting of SRHR literature towards sexual exploitation. Our search strategy includes terminology to capture recognised forms of forced labour, including: forced labour, bonded labour, labour exploitation, involuntary labour, coercive/ed labour, servitude, domestic servitude, and debt bondage. These search terms made up of widely acknowledged forms of, and terminology for forms of, modern slavery and human trafficking should capture studies in high-risk sectors where slavery or trafficking is present. While we understand the reviewer's suggestion to include search terms for high-risk sectors, we have chosen not to include these in our search strategy. We conducted test searches of the suggested high-risk sectors, but these did not yield relevant results. This is likely due to extant literature at the SRHR-MS-HT nexus being overwhelmingly focused on women and girls in sexual exploitation. It is not necessarily surprising that test searches indicated a lack of research on the SRH implications of labour trafficking, particularly in male-dominated industries such as construction, agriculture and fishing. While the team is aware of small bodies of literature on SRHR in agricultural labour, particularly among seasonal workers, this body of literature is not currently focused on those who have been trafficked for that work.
This decision has also been informed by the search strategies used for existing scoping reviews in the field of MS-HT such as:

Care and support during maternity for mothers affected by modern slavery: A scoping review;
Mapping Modern Slavery in the Context of the 2030 Agenda: A Scoping Review Protocol;
Addressing human trafficking through health systems: a scoping review.

We acknowledge the gap in the trafficking proximity clause and thank the reviewer for pointing this out. We have added labour to this clause to ensure we are capturing labour trafficking. We have also added the suggested additional SRH terms as detailed in the updated preliminary Medline search.

8. Gender-inclusive terminology for pregnancy and reproduction: We take on board and completely agree with this feedback. The instances in which gender-exclusive language was used was in reporting statistics from the report of the Guttmacher– Lancet Commission. We have edited the protocol to ensure gender-inclusive terminology in line with a reproductive justice framework.
9. Tension between "no geographical limits" and English-only inclusion: English only inclusion is due to a lack of resources for accurate and reliable translation. We have edited the protocol to make it clear that we are aware this is a key limitation and detailed that we do not have the resources for translation.
10. Patient and public involvement. While working on the search strategy and finalising search terms we did discuss this with our project partners in monthly project meetings. We initially did not view this as PPI because they were general standing meetings in which we also discussed broader project updates. However, after reading the reviewer's feedback on this point, our project partners are survivor-led anti-trafficking and gender-based violence

community organisations and as such are included as PPI. We have edited the protocol to reflect their involvement.
Thank you again to the reviewer for their detailed and helpful feedback. We hope that the revisions made adequately address queries and concerns for publication.

VERSION 2 - REVIEW

**Reviewer**	**1**
**Name**	**Stoklosa, Hanni**
**Affiliation**	**Brigham and Women's Hospital, Massachusetts General Hospital, Emergency Medicine**
**Date**	**21-Aug-2026**
**COI**	

Thank you for the opportunity to review this revised protocol. The authors have engaged seriously with the points raised in my first review. A couple points remain.

1. Data charting in duplicate is still not specified.

Extraction is still unspecified: the charting section describes a pilot on up to ten papers, but does not state whether charting will be carried out by one reviewer, by two independently, or by one with verification by a second. PRISMA-ScR item 10 asks for this explicitly. Please add a sentence stating the approach.

2. Search sensitivity to labour trafficking/forced labour.

I appreciate the addition of the labour term to the trafficking proximity clause and the inclusion of the additional SRH terms, both of which strengthen the strategy.

I would, however, ask the authors to revisit how the search reaches labour exploitation. My concern is less with any particular list of terms than with the absence of vocabulary for the settings in which exploitation occurs and for the workers themselves, as distinct from trafficking as a legal and conceptual category. High-risk-sector terms are one way to address this.

There is precedent for this in the trafficking literature, and the variation in how it has been done is itself informative. See search strategies:

Cockbain E, Bowers K, Dimitrova G. Human trafficking for labour exploitation: the results of a two-phase systematic review mapping the European evidence base and synthesising key scientific research evidence. Journal of experimental criminology. 2018 Sep;14(3):319-60.

Pocock NS, Nguyen LH, Lucero-Prisno DE 3rd, Zimmerman C, Oram S. Occupational, physical, sexual and mental health and violence among migrant and trafficked commercial fishers and seafarers from the Greater Mekong Subregion (GMS): systematic review. Global Health

Research and Policy. 2018;3:28. Note, their health block includes sexual health, STIs, contraception, etc, which suggests SRH evidence in labour trafficking is retrievable when someone sets out to find it. Their occupational-based search terms were roles, fisher, fisherman, seafarer, seaman and boatman. If the authors' pilot searches used industry names alone, that may be part of why they returned little, since papers about exploited workers may name the workers without naming the industry.

Also, in one of the exemplars the authors cite in support of their own decision: the search blocks in Neres et al., "Mapping Modern Slavery in the Context of the 2030 Agenda: A Scoping Review Protocol" (International Journal of Qualitative Methods 2025), include "sweatshop worker", "domestic worker", "sweatshop", "domestic slavery" and "forced domestic work".

The authors have diagnosed the thinness in the literature around labour trafficking accurately in their own manuscript, noting that reviews relevant to SRH in MS-HT have largely focused on the sexual health impacts of commercial sexual exploitation, and again in their response, describing a literature overwhelmingly focused on women and girls in sexual exploitation. Yet forced labour accounts for the largest single share of detected exploitation in the UNODC data this protocol cites. A search that looks only where the literature already concentrates will return a map confirming that concentration, and the gap stays invisible because nothing was designed to detect it.

A protocol, if published in BMJ Open, is read before the search runs and is reused as a template by others working at this intersection. The authors are unusually well placed to set that precedent, having framed this review as the first phase of a reproductive justice programme. Equity in whose exploitation gets looked for is a reasonable thing to expect such a framework to carry into its methods.

Finally, I would add that a null return from a test search is not in itself a reason to omit terms from a scoping review whose stated purpose is to map the extent, nature and distribution of the evidence and the corresponding gaps. If the additional terms retrieve nothing, that absence is a substantive finding, and arguably the finding that most strongly justifies the wider programme of work.

Whether this is best done through sector names, occupational roles, or some combination of the two, is for the authors and their librarian to judge. And if they nonetheless prefer not to expand the search, the decision and its rationale should appear in the protocol.

VERSION 2 - AUTHOR RESPONSE

We would like to thank the reviewer for continuing to engage with our protocol and providing feedback towards further improvements. We have taken the following actions:

Data charting in duplicate is still not specified.

Thank you for pointing out this remaining oversight. We have now added text to the section entitled 'Charting the data' that provides full details of our approach to data extraction.

Search sensitivity to labour trafficking/forced labour

We are happy that the reviewer agrees with the way in which we have added the labour term to the trafficking string and the additional SRH terms. We agree that they have added value to the searches and thank the reviewer for this suggestion.

We have also considered the reviewer's further comments regarding the inclusion of search terms for sectors and occupations with high risks of MS-HT. This further suggestion and rationale given by the reviewer prompted us to sit down and carefully consider the purpose and objectives of our scoping review, as well as to evaluate resources required for expanding the search terms at this stage of our project.

We hope that the reviewer finds the following actions taken satisfactory in addressing their helpful feedback:

Given the opportunity to further discuss and clarify the purpose of our scoping review, we concluded that the review is focused on the sexual and reproductive health issues and challenges faced by individuals who have experienced modern slavery and human trafficking, but not SRH amongst workers in industries at an elevated risk of MS-HT. As such, we concluded that the addition of new occupation/industry-related terms should only be done if it improved sensitivity in the databases for MS-HT contexts. As per the nature of a scoping review, it is our intention to keep the focus of the review broad.

We reviewed the approaches used in the two systematic reviews shared by the reviewer, whilst keeping in mind the difference in their objectives and the nature of systematic reviews as compared with scoping reviews.

Our concern is also that adding sector-specific terms may increase specificity at the expense of sensitivity in the searches. We do not want to restrict identification of studies according to the sector within which exploitation occurred. Although certain sectors are recognized as being at higher risk for exploitation, we also note that sector-specific terminology is inconsistently used in MS-HT literature, with literature variously identifying exploitation by industry, occupation or activity, commodity, or supply chain. Therefore, incorporating sector-specific terms risks both false-negative retrieval through terminology

variation and substantial increase in irrelevant returns.

With all of this in mind – and in the limited time we have given project deadlines and budget constraints - we thought it was prudent to conduct additional exploratory searches using high-risk-sector terminology to ensure robustness of our searches.

We used OVID, which allows comparative searches to be run, and tested the use of over 20 occupation-specific terms. The comparative searches in MEDLINE and EMBASE yielded a total of 713 additional results compared to searches run on 1st September as per the stated timeline. The titles and abstracts of the updated results were individually screened by two reviewers. However, it was found that none of the additional returns met inclusion criteria. We feel that, due to the complexity of high-risk sector terminology, the sexual and reproductive health of workers in high-risk sectors likely justifies its own separate scoping review.

We have therefore decided not to include additional terms relating to high-risk sectors. While we recognise the value of such terms for reviews focused specifically on labour exploitation and the settings in which it occurs, our review is focused on legally defined and recognised forms of MS-HT, rather than on sectors or occupational settings in which MS-HT may be more likely to occur. Expanding the search in this way would also require substantial revisions to the search strategies across all eight databases and would likely generate a considerable number of additional records, many of which would fall outside the review's inclusion criteria, as per the findings from our recent exploratory searches. Given the scope and objectives of the review, as well as the available timeline and resources, we do not consider this expansion necessary for this particular review.

Nonetheless, we want to make sure that our search is sensitive enough to forms of labour exploitation in MS-HT beyond that of sexual exploitation. As such, we returned to our original searches conducted after submission of the first version of our protocol on 6th May 2026, and before we received the first round of feedback on 28th July 2026. From those searches, run in June 2026, eight papers that met our inclusion criteria contained extractable date on or information about those who have experienced labour exploitation in MS-HT.

Having run the updated searches on 1st September that better ensures we are catching 'labour' in proximity to 'trafficking' as well as previously included labour exploitation terms, we are confident that the search strings are picking up literature at the intersection of labour trafficking, forced labour, and labour exploitation and SRH.

To further ensure coverage of labour exploitation beyond sexual exploitation, however, we have also taken the decision to further expand the grey literature search to include the websites of labour-specific anti-trafficking/slavery organisations: the International Labour Organization, Focus on Labour Exploitation, as well as Walk Free Foundation, and the International Organization for Migration.

We do also recognise the importance of the context within which exploitation is occurring and as such, we have retained sector/industry as a variable for data extraction and analysis, rather than a pre-requisite for study retrieval.

As per the reviewer's suggestion, we have included this decision and its rationale in the updated protocol.
