# Supplementary material for: Sexual and reproductive health impacts of modern slavery and human trafficking: a scoping review protocol
Source: BMJ Open. 2026 Oct 7;16(10):e122246. doi: 10.1136/bmjopen-2026-122246 (PMC13647010; doi:10.1136/bmjopen-2026-122246)
Supplement: Supplementary file 2 [file bmjopen-16-10-s001.docx]

Appendix

*Preliminary search terms for OVID Medline*

Exp Human Rights Abuses/ or Enslaved Persons or (human* or sex* or people or person* or wom#n* or female* or child* or marriage or labo?r) adj3 traffic*) or (force* or bonded or exploit* or involuntar* or coerc*) adj3 (labo?r* or servitude or marriage* or sex* or pregnanc* or prostitut* or financial* or debt*) or slave* or bondage* or "domestic servitude" or "debt bondage").mp.

AND

Sexual Health/ or Reproductive Health/ or exp Sexually Transmitted Diseases/ or exp Reproductive Medicine/ or exp Women’s Health/ or exp Pregnancy/ or exp Abortion, Induced/ or exp Reproductive Health Services/ or exp Contraception/ or Menstruation/ or Obstetrics/ or exp Maternal Health Services/ or Maternal Health/, exp Sterilization, Reproductive/ or Family Planning Services/ or (sex* or reproduct* or matern* or "family planning") adj2 (heath* or care or service* or clinic* or educat* or counselling) or (abortion* or sterilis* or steriliz* or contracepti* or pregnanc* or "birth control" or "sexually transmitted disease" or STD or "sexually transmitted infection*" or "sexually transmitted virus*" or STD or STI or "wom#n* health*" or obstetric* or gynaecol*menstrua* or "menstrual health" or "reproductive coerc*" or "reproductive violence").m.p.

limit 24 to yr="2000 -Current"
